# The Verbal Interaction Social Threat Task: A New Paradigm Investigating the Effects of Social Rejection in Men and Women

**DOI:** 10.3389/fnins.2019.00830

**Published:** 2019-08-07

**Authors:** Sanne Tops, Ute Habel, Ted Abel, Birgit Derntl, Sina Radke

**Affiliations:** ^1^Department of Psychiatry, Psychotherapy and Psychosomatics, Faculty of Medicine, RWTH Aachen University, Aachen, Germany; ^2^Jülich Aachen Research Alliance – BRAIN Institute I: Brain Structure–Function Relationships: Decoding the Human Brain at Systemic Levels, Research Center Jülich GmbH and RWTH Aachen University, Aachen, Germany; ^3^Department of Molecular Physiology and Biophysics, Iowa Neuroscience Institute, Carver College of Medicine, The University of Iowa, Iowa City, IA, United States; ^4^Department of Psychiatry and Psychotherapy, Medical School, University of Tübingen, Tübingen, Germany

**Keywords:** social-evaluative threat, rejection, cortisol, social stress, verbal communication, VISTTA

## Abstract

In recent years, digital communication and social media have taken an indispensable role in human society. Social interactions are no longer bound to real-life encounters, but more often happen from behind a screen. Mimicking an online communication platform, we developed a new, fMRI compatible, social threat paradigm to investigate sex differences in reactions to social rejection. During the Verbal Interaction Social Threat Task (VISTTA), participants initiate 30 short conversations by selecting one of four predefined opening sentences. Two computerized interlocutors respond to the opening sentence mostly with negative comments and rejections toward the participant, which should induce social-evaluative threat. Physiological and subjective responses were measured, before, during, and after the VISTTA in 61 (29 male and 32 female) first year students who received either mostly negative (*n* = 31; threat group) or neutral comments (*n* = 30; control group). Two-level behavioral validation included social threat-induced mood changes in participants, and interlocutor evaluation. The latter consisted of multiple variables such as “willingness to cooperate” after every conversation, an overall fairness evaluation of interlocutors, and evaluations per reaction indicating how positive or negative it was received. We acquired additional physiological measures including cortisol assays via saliva samples, heart rate, and blood pressure. Confirming our hypotheses, peer rejection and exclusion during the VISTTA led to less willingness to cooperate and lower fairness evaluation of interlocutors. It also induced feelings of anger and surprise and lower happiness in the social-threat group. Women showed overall higher emotion ratings compared to men. Contrary to our *a priori* hypothesis, the VISTTA did not induce cortisol and heart rate increases. However, the stable cortisol response in women in the threat group does not follow the circadian decline and might reflect an endocrinological response. The decline in cortisol response in men in both the threat and control group could indicate faster habituation to the VISTTA. Taken together, these findings indicate effects of social-evaluative threat on a behavioral level, and more moderate effects on the emotional and physiological level. Sex differences in affective and cortisol responses may indicate that women are more susceptible for the social-evaluative threat than men. With a realistic implementation of verbal, interactive, and social components, the VISTTA is designed as an fMRI paradigm that can be applied to elucidate the neural representation of social-evaluative threat.

## Introduction

With the increasing influence of social media and online communication platforms, digital communication has taken a vital role in current society. With this development, social interaction more often happens from behind a screen, rather than in real life. Interactions that involve rejection, exclusion, and negative evaluation can lead to a lower self-esteem and acceptance (Dickerson, 2008). This can also lead to a set of physiological responses including activation of one of the main biological responses to stress: the hypothalamus–pituitary–adrenal gland (HPA) axis (Mason, 1968), leading to an increased production of cortisol by the adrenal glands (Lupien et al., 2009). In the initial definition of stress by Selye (1950), “mere emotional stimuli” were considered negligible in comparison to physical variables such as physical trauma, heat, and fasting. Emotional stimuli, that is, conditions involving novelty, uncertainty, unpredictability, and anticipation of something previously experienced as unpleasant, however, may challenge one’s capacity to cope with the situation, which will be experienced as a burden and distress.

As proposed by Mason (1968), emotional stimuli such as social evaluation and exclusion can also trigger the stress response. This idea has been confirmed by more recent studies (Williamson et al., 2018) investigating the effect of social exclusion on cardiovascular and affective responses in response to a social evaluative stressor. Excluded participants showed increased cardiovascular and anxiety responses to the stressor. Included participants reported similar increases in anxiety, but cardiovascular responses did not change. Social evaluation functions as a stressor through the salience of negative judgment, and the threat that it poses to maintaining self-esteem and social status. Uncontrollable and social-evaluative elements of a psychological stressor have been shown to increase cortisol and blood pressure (Dickerson and Kemeny, 2004). The threat is specific but common; several studies have indicated that cortisol rises after social evaluation in various settings such as public speaking, paced auditory serial addition test, and mental arithmetic under time pressure (Kirschbaum et al., 1993; Bibbey et al., 2015; Smith and Jordan, 2015; Dahm et al., 2017).

The stress response is not universal. Differences between men and women responding to various stressors have been well documented. Multiple underlying factors have been identified, often divided into biological and social factors. The menstrual cycle and oral contraceptives (OCs) have been found to affect the stress response. During the follicular phase of the menstrual cycle, the cortisol response is attenuated compared to the luteal phase (Villada et al., 2017), possibly explained by higher levels of progesterone and estrogen in the luteal phase (Gordon and Girdler, 2014). OC use has a dampening effect on the stress response. A meta-analysis based on 34 studies by Liu et al. (2017) reported lower salivary cortisol in women on OCs compared to women not on OC both at baseline and peak following the Trier Social Stress Test (TSST) but not during the recovery phase. When comparing men to women on OC and men compared to women not on OC, they found no differences in salivary cortisol at baseline between the sexes, but reported higher cortisol levels in men during peak and recovery compared to women on OC. In addition to biological factors, gender and socialization seem to affect the stress response in men and women differently (Pruessner, 2018). When being subjected to psychosocial stress, social support from a partner dampens the cortisol response in men, women on the other hand respond more strongly with their partner around (Kirschbaum et al., 1995). Comparing an achievement stressor with a social rejection stressor, Stroud et al. (2002) showed increases in cortisol in men for the former and in women in the latter Task. This suggests that men are more sensitive to competitive and achievement aspects of a situation, and that women are more affected by social components that can affect their social standing within a group. They did not differentiate between sex and gender and only included women who were not on OC. There is, however, empirical evidence showing increased cortisol and testosterone levels in women in anticipation of a rugby match, whereby postgame levels of these hormones were higher than pregame levels (Bateup et al., 2002). The testosterone rise was associated with team bonding and aggressiveness and the cortisol change was positively related to the level of challenge of the opponent. These findings provide evidence that not only men, but women too are sensitive to competitive aspects of a situation and that it is reflected in their endocrinological response. A study applying an adjusted TSST, whereby the audience during the 5 min speech was behind a one-way mirror so participants could not see them, yielded sex-specific results. Men reported comparable cortisol levels, whereas women showed no response when they could not see the audience (Andrews et al., 2007; Wadiwalla et al., 2010). This sex-based difference paved the way for follow-ups investigating the influence of gender identity on the stress response. Sex refers to physiological differences in the gonads, sex hormones, external genitalia, and internal reproductive organs. Gender on the other sided refers to social, environmental, cultural, and behavioral factors that affect someone’s self-identity (Clayton and Tannenbaum, 2016). To differentiate between the effects of sex and gender identity, four groups were subjected to the adjusted TSST: male gender identity with male sex, female gender with female sex, male gender with female sex, and female gender with male sex. The *cis*-gendered groups replicated previous results. However, subjects with female gender identity combined with male sex did not respond to the Task, whereas subjects with male gender identity and female sex responded like *cis*-gendered males with an increased cortisol response (Pruessner, 2018). Future studies examining the effect of OC on women with female vs. male gender identity could give more insight which factor dominates the stress response. These results emphasize the importance of gender identity in explaining differences between men and women.

Inducing social-evaluative threat commonly involves evaluation and judgment by others. Numerous stress paradigms comprise both social evaluation and performance, such as the TSST, Montreal Imaging Stress Task (MIST), and ScanStress (Kirschbaum et al., 1993; Dedovic et al., 2005; Dahm et al., 2017, respectively). Evaluation through rejection/exclusion has often been examined using the Cyberball paradigm (Williams et al., 2000). A modified version of the Cyberball Task, with exclusion based on negative performance evaluation, proved to increase subjective stress (Wagels et al., 2017). Due to the mild nature of exclusion in the original Cyberball, however, cortisol increases are not consistently found (Zöller et al., 2010; Seidel et al., 2013; Gaffey and Wirth, 2014; Radke et al., 2018). Similarly, the Yale Interpersonal Stressor (YIPS) is a well-established way to induce social-evaluative threat, whereby participants are excluded during the course of a real-life conversation with two confederates (Stroud et al., 2002; Zwolinski, 2008). While Stroud et al. (2000, 2002) reported a cortisol increase following the YIPS, others also relying on the YIPS failed to elicit a cortisol response (Linnen et al., 2012). Following recent developments in computer-mediated communication, a novel, exclusion-based paradigm, “Ostracism Online,” mimics a social media environment to induce social exclusion (Wolf et al., 2014). Here, participants can receive “likes” from others on a short introduction they wrote about themselves; in the exclusion condition they, however, receive only one “like” from 11 other group members (Wolf et al., 2014). Ostracism Online has been validated using the Need-Threat Scale (van Beest and Williams, 2006) and has been reported to induce increased self-ratings in the extent to which participants felt bad, unfriendly, angry, and sad following exclusion compared to including conditions. Mimicking more realistic online communication, Donate et al. (2017) developed a chatroom Task whereby participants can ask questions to and answer questions from two confederates in a yes or no format. Participants in the inclusion condition are asked a question in 33% of the rounds (equal to the confederates), compared to only 15% in the exclusion condition. The results revealed that exclusion led to increased anger and higher levels of self-pain feelings, namely feeling tortured and hurt. It is important to note that both paradigms have been validated using self-ratings only.

Overall, responses to social-evaluative threat can be assessed on various levels. Performance oriented paradigms, like the MIST, ScanStress, and TSST, confirmed their validity with physiological measures using cortisol assays. Exclusion-related paradigms such as Cyberball and Ostracism Online mainly focused on subjective ratings of stress and mood to indicate an emotional effect.

There is, however, no fMRI compatible paradigm available yet that combines social-evaluative threat with social media- or online communication. We have therefore designed the “Verbal Interaction Social Threat Task” (VISTTA) suitable for investigating the direct neural representation of social-evaluative situations and responses. This study is the first to investigate the possibilities and implications of the VISTTA, aiming to validate it as a social threat induction method. Considering the scope of the current study, additional research will have to be done to have a broader understanding of the domains affected by the VISTTA. Verbal communication is central to this new paradigm that bears a strong resemblance to online chatting. The increasing influence of social media and online communication platforms comes with an increase in the number of cases of cyberbullying. The Cyberbullying Research Center in the United States reported that on average 28% of all middle and high school students, who participated in different studies between 2007 and 2016, have been the victim of cyberbullying (Patchin, 2016). The VISTTA mimics an online communication environment with two interlocutors. It has a realistic implementation of verbal, interactive, and social components and can be deployed to gain valuable insights in the above-described social interactions. Participants are told they will do a cooperation Task with the interlocutors at the end of the VISTTA and are asked after every conversation to rate how much they like to cooperate with them. We expect the VISTTA to elicit a behavioral, emotional, and a physiological response. Lower subjective ratings with regard to the cooperation Task and a more negative mood indicate a behavioral and emotional effect, respectively. We also expect to find elevated cortisol levels, and increased heart rate over the course of the paradigm. Based on the above-described sex differences, we hypothesized to find larger effects in females than in males.

## Materials and Methods

### Ethics Statement

The local ethics committee at the Medical Faculty of RWTH Aachen University approved the current study. The experimental protocol was carried out in accordance with the provisions of the World Medical Association Declaration of Helsinki.

### Participants

Sixty-one healthy first year students (29 males, *M*_age_ = 19.9, *SD* = 1.6, 32 females, *M*_age_ = 19.85, *SD* = 1.2; sex was defined by self-report) who were all fluent in German, participated in this experiment. When discussing males/females, we refer to the sex that is reflected physiologically by the gonads, sex hormones, external genitalia, and internal reproductive organs (Clayton and Tannenbaum, 2016). To ensure that all participants were in a new social environment without an established social network, we only included students who had recently moved to Aachen and did not switch studies. Further inclusion criteria were: age of 18–30 years, right-handedness, no metabolic illnesses (hypertension; lung-, brain-, and kidney diseases; diabetes mellitus; and drug dependence), no neurological or psychiatric disorders (determined with Structured Clinical Interview for DSM-IV; Fydrich et al., 1997), no medication use, and no pregnancy in women. Only women who took hormonal contraceptives via the pill were included. We made this choice to control for hormonal fluctuations and interpersonal differences in the menstrual cycle (Liu et al., 2017). Participants were told to refrain from alcohol 24 h, eating 2 h, and coffee 3 h prior to the experiment. All experimental sessions took approximately 2 h and took place between 1.30 pm and 6.30 pm to control for the circadian rhythm in cortisol. All participants gave written informed consent and received €20, as monetary compensation.

### Paradigm – Social-Evaluative Threat

The VISTTA is partially based on the YIPS (Stroud et al., 2000). The YIPS uses real confederates that are trained to exclude the participant from the conversation, whereas the VISTTA is a variation that employs a digital communication platform. The Task simulates an online communication environment in which the participant is led to believe he/she is communicating with two peers, one male (Daniel) and one female (Julia).

**FIGURE 1:**
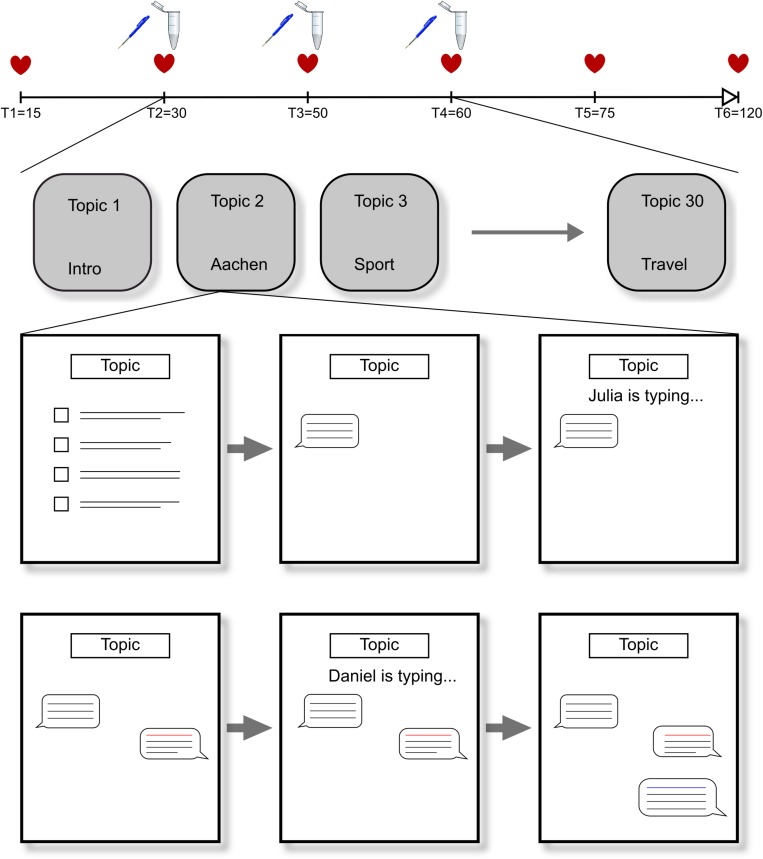
Overview of the experiment. The participant selects an opening sentence, which is then presented in the chatbox. The two interlocutors respond consecutively. The last page including both their responses is presented for 10 s to give participants enough time to read and think about them.

**FIGURE 2:**
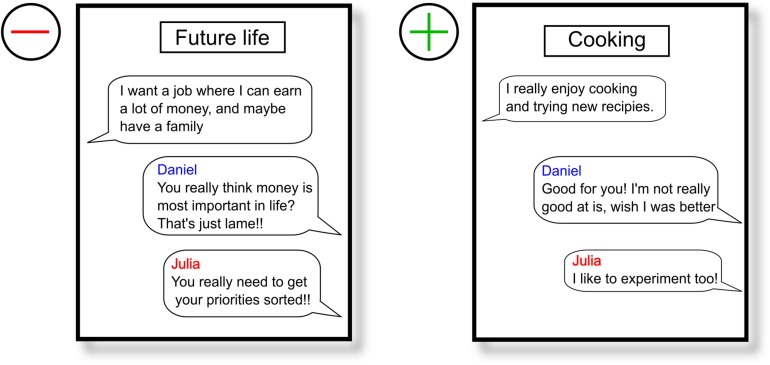
Two examples of conversations, a negative on the left, and a positive on the right. The blue line represents a response by the male interlocutor (Daniel) and the red line corresponds with the female interlocutor (Julia).

During 30 short conversations, which the participant initiates by selecting one of the four presented opening sentences, the two computerized interlocutors respond mostly with negative comments and rejections toward the participant. This approach should induce feelings of social rejection and exclusion and induce feelings of social stress in the participants. The participants had 40 s to select an opening sentence by pressing button 1, 2, 3, or 4 on the keyboard. If they did not select an option, the first sentence was automatically selected. We created unique reactions per opening sentence, in a way that they did not contradict over the course of the experiment. After selecting an opening sentence, a chat box was shown whereby “… is typing” was shown when the interlocutors were supposedly typing there response. Depending on the length of the response, the duration of “… is typing” varied in length with longer presentation times for longer responses (ranging from 4 to 8 s) (see Figure 1 for an overview of the VISTTA). In order to create a within-subject control condition and to make it more credible that participants were chatting with two actual people, they also received neutral to positive reactions in 10 out of 30 conversations. Reactions from interlocutors were both either positive or negative, so that acceptance and rejection would take place in the same set of topics for all participants. Two examples are presented in Figure 2. This experimental group, from here on referred to as “threat group,” was compared to a control group that only received neutral/positive reactions. The 20 topics with dismissive comments were changed so that the interlocutors replied with agreement and consent. A pilot study among Ph.D. and master students (*n* = 15 for threat condition, *n* = 11 for control condition) showed that all negative responses were rated significantly more negative than all positive responses (*p* ≤ 0.009), except one that showed only a trend toward significance (*p* = 0.052) (see Supplementary Table 1 for all opening sentences and corresponding reactions). We created two pseudorandom orders of topics that were randomly assigned to the participants to rule out any possible confounding effects of order. Each block of 10 conversations contained the same set of topics in both versions. All responses were created to match the opening sentences. The experiment started with a practice round to familiarize the participants with the structure of the paradigm.

### Procedure

As part of the cover story, participants were told that the experiment was about initiating social interactions between students via online communication and that the two interlocutors were each in a separate room nearby. They were also led to believe they had to do a cooperation Task with the two others after the VISTTA and that the height of their monetary reward depended on how well they cooperated. It was therefore preferable if they maintained a good bond with the interlocutors. To reinforce the cover story, participants were told to be punctual, because the experiment was conducted together with two other students. They could not meet the “others,” as the goal of this study was said to investigate online communication. During the experiment, the investigator left the participant three times to check whether the “others” were ready to start and if everything went as planned; first before the start of the VISTTA (T2), in the short break after 20 conversations (T3), and directly after finishing the VISTTA (T4). Mood and physiological measures were also acquired at these time points. During the debriefing at the end of the experiment, all participants were asked about their experience with regard to the confederates, and whether they believed they were communicating with two real people. Five participants reported they did not believe the two interlocutors were real. Exploratory analyses whereby non-believers were excluded yielded a similar pattern of results. Final analyses were therefore conducted on the whole sample. As the study was conducted in Aachen, Germany, all opening sentences and responses were in German (see Supplementary Table 1 for the English translation).

### Social-Evaluative Threat Measures

#### Trait Measures

We acquired a set of personality questionnaires covering stress coping mechanisms [Coping Inventory for Stressful Situations (CISS); Endler and Parker, 1990], anxiety {State-Trait Anxiety Inventory [STAI(T)]; Spielberger et al., 1983, Liebowitz Social Anxiety Scale; Liebowitz, 1987}, primary appraisal secondary appraisal (PASA; Gaab, 2009), rejection sensitivity [Rejection Sensitivity Questionnaire (RSQ); Berenson et al., 2009], social network questionnaire (Linden et al., 2007), stress processing (Stressverarbeitungsfragebogen; Janke and Erdmann, 1997), and intelligence [Wortschatztest (WST); Schmidt and Metzler, 1992].

#### Subjective Ratings

The subjective experience of social evaluative threat was assessed on three distinct levels. First, at the end of every conversation, participants were asked to rate the extent to which they wanted to cooperate with the two interlocutors on a Scale from 1 to 5, with 1 being “not at all” and 5 “very much.” Second, after finishing the VISTTA, participants answered open questions how they experienced the interaction and how they felt to not have met their interlocutors. They also rated on a Scale from 1 to 8 how fair they thought the “others” were (1 being “not fair at all,” 8 being “very fair”). Third, in an additional, reaction rating Task, participants were presented with all reactions (2 reactions per topic, total of 60 reactions) they had received during the VISTTA. For each reaction separately, they indicated to what extend they experienced that reaction as positive or negative in regard to their opening sentence (on a Scale from 1 to 5 with 1 being “very negative” and 5 “very positive”).

#### Emotional and Physiological Responses

Mood was measured repeatedly using the Emotional Self-Rating (ESR; Weiss et al., 1999) and the Positive and Negative Affect Scale (PANAS) at T2, T3, and T4 (Watson et al., 1988).

Salivary cortisol levels, heart rate, and blood pressure were repeatedly measured throughout the experiment (see Figure 1 for overview). Saliva samples were taken using SaliCaps, to measure cortisol (IBL International, Hamburg, Germany). Saliva samples were taken at the start of the VISTTA (T2), in the short break after 20 conversations (T3), and directly after finishing the VISTTA (T4). Sampling time varied among participants, but was not timed. The samples were stored at −30°C until they were analyzed by the Dresden LabService (Germany). Samples were analyzed in duplicate and the average was used in subsequent analyses. Cortisol concentrations were measured using Luminescence Immunoassays with high sensitivity (Immuno-Biological Laboratories GmbH, Hamburg, Germany), with intra-assay and inter-assay coefficients of <8%. Heart rate and blood pressure were acquired via an automatic blood pressure monitor with arm cuff (Intellisense, OMRON, Germany) at six time points (minutes after onset) throughout the experiment (T1 = 15, T2 = 30, T3 = 50, T4 = 60, T5 = 75, T6 = 120).

### Statistical Analyses

All analyses were performed using SPSS 25 (IBM Corp., Armonk, NY, United States). The alpha level was set to 0.05 and Greenhouse–Geisser correction was applied when necessary. *Post hoc* pairwise comparisons were Bonferroni corrected.

#### Trait Measures

All scores except PASA and Social Network were normally distributed. PASA and Social Network were logarithmically transformed to meet the criterion of normal distribution. Separate 2 × 2 ANOVAs were conducted for each of the personality questionnaires, with Group (threat, control) and Sex (male, female) as between-subject factors.

#### Subjective Ratings

Ratings on the willingness to cooperate were averaged for positive/neutral and negative reactions separately. Subsequently, a 2 × 2 × 2 ANOVA was conducted, with Valence (positive or negative reactions) as within-subject factor and Group and Sex as between-subjects factors. A similar analysis was used for the fairness rating, without Valence as a within-subject factor (i.e., a 2 × 2 ANOVA).

Ratings for the individual reactions all deviated from normal distribution (Kolmogorov–Smirnov was significant). Moreover, the subset of reactions presented to the participant depended on the choice of opening sentence, which led to a unique combination of reactions for every participant. Hence, individual reactions could not be directly compared between conversations. The ratings per reaction (one from each interlocutor) were combined into a mean score indicating the overall positivity/negativity of the reaction-pair per conversation. For these reasons, these data were analyzed using generalized estimating equations (GEEs). This mean rating was entered as dependent variable in the full model of the GEE analysis with Topic Valence (two levels: positive, negative) as within-subject factor and Group (threat, control) and Sex (male, female) as between-subject factors. Subjects were modeled as random effects and all factors as fixed effects.

#### Emotional Responses

Repeated measures ANOVAs with *post hoc* pairwise comparisons were conducted for PANAS, with positive and negative mood as subscales, with Time (T2, T3, T4) as within-subjects factor and Group (threat, control) and Sex (male, female) as between-subjects factors.

A similar analysis as for the reaction ratings was performed for the ESR Scales (anger, disgust, happiness, fear, sadness, surprise) as they deviated from normal distribution. The GEE analysis was designed with Emotion (six levels: anger, disgust, happiness, fear, sadness, surprise) and Time (T2, T3, T4) as within-subject factors and Group (threat, control) and Sex (male, female) as between-subject factors. Subjects were modeled as random effects, and all factors were included as fixed effects. To test for differential effects of the two VISTTA versions, only interactions involving the factor Group were entered in the model (as fixed effects).

#### Physiological Responses

Cortisol was acquired three times and heart rate and blood pressure six times. Cortisol values were not normally distributed and hence logarithmically transformed. All analyses were computed based on the transformed data. Repeated measures ANOVAs were conducted for cortisol, heart rate, systolic and diastolic blood pressure, with Time as within-subjects factor and Group and Sex as between-subjects factors.

## Results

### Trait Measures

There were no differences between groups or sexes regarding the personality questionnaires, after correcting for multiple comparisons. Before correction, scores on trait anxiety (STAI-T; *p* = 0.041) and Task-oriented coping (CISS_Task; *p* = 0.013) were significantly higher for men than women, whereas scores on avoidance-oriented coping (CISS_avoidance; *p* = 0.009), and social anxiety (Liebowitz_anxiety: *p* = 0.014), were higher for women compared to men. Both, however, did not survive the corrected alpha level that was set to 0.0029. All other comparisons were not significant. The descriptive statistics of all questionnaires are included in Supplementary Table 2.

### Subjective Ratings

#### Willingness to Cooperate and Perceived Fairness

**FIGURE 3:**
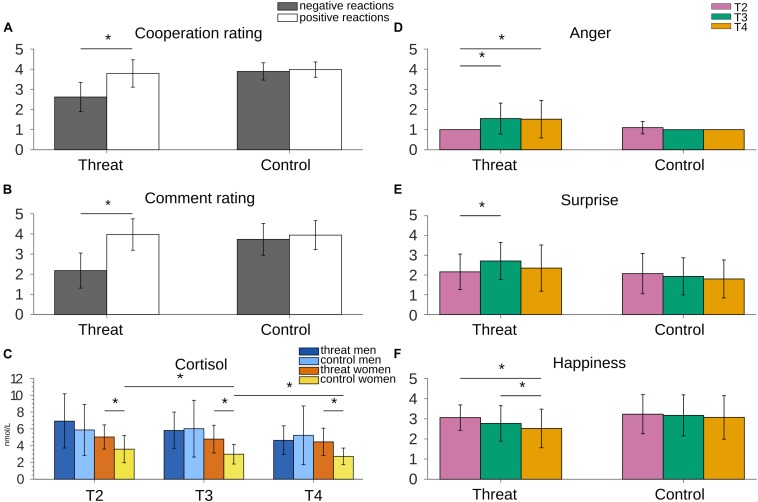
**(A)** Willingness to cooperate was lower after negative reactions compared to positive reactions. As expected, no difference was present in the control group. **(B)** Negative reactions in the threat version of the VISTTA were rated significantly lower than reactions to the same topics in the control version. Also within-subject they were rated lower than the positive reactions in 10 topics. **(C)** Cortisol stayed stable in women in the threat group, but decreased in the control group. Men showed decreasing cortisol levels in both groups. For illustrative purposes only significant differences between women in both groups and the decay in the women control group are marked with an asterisk. **(D)** Anger increased from T2 to T3 in the threat group, compared to unchanged anger scores in the control group. **(E)** The threat version led to an increased score of surprise, whereas this score stayed stable in the control group. **(F)** Participants in the threat group reported decreased levels of happiness. The control version did not elicit such a decrease. Raw values were used to create the graphs, although some analyses used transformed scores. Error bars represent standard deviations. Asterisks indicate significant differences with *p* < 0.05.

As expected, participants who took part in the threat group were less willing to cooperate with the interlocutors than participants who received only neutral/positive reactions during the Task. This was shown by a main effect of Group [*F*(1,57) = 37.254, *p* ≤ 0.001, $\eta_{\text{p}}^{2}$ = 0.395], whereby the overall “willingness to cooperate” was lower in the threat group (*M* = 3.21, *SD* = 0.53) than in the control group (*M* = 3.94, *SD* = 0.37) (*p* ≤ 0.001). We also found a main effect of Valence [*F*(1,57) = 49.033, *p* ≤ 0.001, $\eta_{\text{p}}^{2}$ = 0.462], showing higher “willingness to cooperate” after positive (*M* = 3.88, *SD* = 0.55) than after negative (*M* = 3.25, *SD* = 0.87) reactions. A Valence ^*^ Group interaction [*F*(1,57) = 35.082, *p* ≤ 0.001, $\eta_{\text{p}}^{2}$ = 0.381] revealed a group difference for negative reactions, with lower cooperation ratings in the threat group compared to the control group (*p* ≤ 0.001). No group difference was found for positive reactions (*p* = 0.453). “Willingness to cooperate” also significantly differed within-subjects in the threat group, with higher ratings after positive comments than after negative comments (*p* ≤ 0.001) (Figure 3A). No Valence effect was present in the control group (*p* = 0.453), as all reactions were neutral/positive. “Willingness to cooperate” did not differ between sexes, regardless of valence (*p* = 0.438) (see Table 1 for means per group and emotion).

**TABLE 1:** Overview of means of subjective and emotional responses in men and women in the threat and control group.

	**Control men (*N* = 14)**	**Control women (*N* = 16)**	**Threat men (*N* = 15)**	**Threat women (*N* = 16)**
**Subjective ratings**				
Cooperation positive	3.85 (0.27)	4.1 (0.43)	3.81 (0.78)	3.78 (0.59)
Cooperation negative	3.74 (0.34)	4.02 (0.47)	2.69 (0.78)	2.55 (0.7)
Fairness	7.36 (1.03)	7.5 (1.26)	4.1 (1.49)	4.34 (1.17)
Comment positive	4.01 (0.75)	3.85 (0.69)	3.79 (0.84)	4.1 (0.71)
Comment negative	3.83 (0.81)	3.62 (0.75)	2.16 (0.89)	2.19 (0.85)
**Emotional responses**				
PANAST2pos	29.86 (6.5)	31.5 (5.93)	30.62 (3.78)	30.75 (5.12)
PANAST3pos	29 (5.92)	31.06 (7.09)	29.08 (6.03)	29.63 (6.86)
PANAST4pos	25.86 (6.74)	30.38 (7.2)	27.86 (4.61)	28.44 (8.07)
PANAST2neg	11.64 (1.86)	12.81 (3.04)	12.29 (2.61)	11.38 (1.78)
PANAST3neg	11.21 (2.26)	11.25 (1.61)	13.2 (3.41)	11.81 (2.76)
PANAST4neg	11.36 (1.98)	11.06 (1.48)	12.67 (3.2)	11.94 (3.77)
AngerT2	1.07 (0.27)	1.13 (0.34)	1.00 (0.00)	1.00 (0.00)
AngerT3	1.00 (0.00)	1.00 (0.00)	1.60 (0.82)	1.50 (0.73)
AngerT4	1.00 (0.00)	1.00 (0.00)	1.47 (0.83)	1.56 (1.03)
DisgustT2	1.00 (0.00)	1.06 (0.25)	1.07 (0.26)	1.00 (0.00)
DisgustT3	1.00 (0.00)	1.00 (0.00)	1.07 (0.26)	1.00 (0.00)
DisgustT4	1.00 (0.00)	1.00 (0.00)	1.00 (0.00)	1.00 (0.00)
HappinessT2	2.93 (0.92)	3.50 (0.97)	3.00 (0.54)	3.13 (0.72)
HappinessT3	2.71 (1.07)	3.56 (0.81)	2.53 (0.83)	3.00 (0.89)
HappinessT4	2.50 (1.02)	3.56 (0.89)	2.27 (0.80)	2.75 (1.07)
SadnessT2	1.00 (0.00)	1.19 (0.40)	1.07 (0.26)	1.00 (0.00)
SadnessT3	1.07 (0.27)	1.06 (0.25)	1.07 (0.26)	1.00 (0.00)
SadnessT4	1.00 (0.00)	1.06 (0.25)	1.07 (0.26)	1.06 (0.25)
SurpriseT2	2.00 (0.96)	2.13 (1.09)	2.33 (0.98)	2.00 (0.82)
SurpriseT3	1.79 (0.70)	2.06 (1.12)	2.73 (0.96)	2.69 (0.95)
SurpriseT4	1.79 (0.80)	1.81 (1.11)	2.33 (1.23)	2.38 (1.15)
FearT2	1.00 (0.00)	1.19 (0.40)	1.07 (0.26)	1.06 (0.25)
FearT3	1.00 (0.00)	1.13 (0.34)	1.07 (0.26)	1.06 (0.25)
FearT4	1.00 (0.00)	1.00 (0.00)	1.07 (0.26)	1.06 (0.25)

*Cooperation positive and – negative (willingness to cooperate after positive and negative reactions, respectively), comment positive and – negative (how positive or negative both reactions from interlocutors were experienced by participants).*

Interlocutors in the threat group (*M* = 4.22, *SD* = 1.32) were rated significantly less fair than interlocutors in the control group (*M* = 7.43, *SD* = 1.14) [*F*(156.34,1.56) = 1319.08, *p* ≤ 0.001, $\eta_{\text{p}}^{2}$ = 0.637]. There was no main effect of Sex on the willingness to cooperate and no significant interaction including Sex emerged (all *p* ≥ 0.579).

#### Comment Ratings

The GEE analysis for the comment ratings showed a main effect of Group [Wald-χ^2^(1) = 87.0, *p* ≤ 0.001], whereby reactions were rated lower (more negative) in the threat group (*M* = 2.77, *SD* = 1.19) than the control group (*M* = 3.80, *SD* = 0.77). We also found a main effect of Topic Valence [Wald-χ^2^(1) = 221.5, *p* ≤ 0.001], with lower ratings for negative comments (*M* = 2.94, *SD* = 1.14) compared to neutral/positive comments (*M* = 3.95, *SD* = 0.75). Again, there was no main effect of Sex (*p* = 0.931). Two interactions were found significant, i.e., Group ^*^ Sex [Wald-χ^2^(1) = 47.0, *p* = 0.031] and Group ^*^ Topic Valence [Wald-χ^2^(1) = 137.9, *p* ≤ 0.001]. No other interactions were significant (*p* ≥ 0.221). *Post hoc* analyses for the Group ^*^ Sex interaction showed that both men and women in the threat group rated the comments overall as more negative than the participants in the control group (*p* ≤ 0.001). Within the threat group, men tended toward lower ratings (more negative) than women (*p* = 0.093). There was no difference in ratings between men and women in the control group (*p* = 0.152). Decomposing the Group ^*^ Topic Valence interaction showed that reactions in the 20 negative topics in the threat group were rated more negative than neutral/positive reactions to the same topics in the control group (*p* ≤ 0.001). The reactions to the 10 neutral/positive topics that were the same for both groups were rated equally (*p* = 0.905) (Figure 3B).

### Emotional Responses

#### Positive and Negative Mood

The VISTTA led to a significant decrease in positive mood over time, that is, there was a main effect of Time for positive mood [*F*(1.793,98.613) = 14.651, *p* ≤ 0.001, $\eta_{\text{p}}^{2}$ = 0.210]. Pair-wise comparisons showed a general decrease between T2−T3 and T2−T4 (*p* ≤ 0.001). We did not find a main effect of Time for negative mood (*p* = 0.545). A Time ^*^ Group interaction did occur for negative mood [*F*(1.558,98.613) = 6.012, *p* = 0.007, $\eta_{\text{p}}^{2}$ = 0.099], but not positive mood (*p* = 0.677). *Post hoc* analyses showed that negative mood decreased only in the control group between T2−T3, that is, from the start of the Task until the break. Negative mood did not change over time in the threat group (*p* ≥ 0.272). No other effects or interactions were found significant (*p* ≥ 0.197).

#### Emotional Self-Rating

The GEE analysis for the ESR revealed significant main effects of Emotion [Wald-χ^2^(5) = 532.1, *p* ≤ 0.001], Sex [Wald-χ^2^(1) = 4.63, *p* = 0.031], and Time [Wald-χ^2^(2) = 7.66, *p* = 0.022].

There were significant interactions of Group ^*^ Time [Wald-χ^2^(2) = 6.8, *p* = 0.033], and Group ^*^ Emotion [Wald-χ^2^(5) = 15.98, *p* = 0.007]. In addition, there were significant three-way interactions of Group ^*^ Emotion ^*^ Time [Wald-χ^2^(17) = 43.4, *p* ≤ 0.001], and Group ^*^ Sex ^*^ Emotion [Wald-χ^2^(10) = 19.01, *p* = 0.040].

The main effect of Emotion was due to significantly higher ratings for happiness (*M* = 2.97, *SD* = 0.95) than for all other emotions, followed by surprise (*M* = 2.17, *SD* = 1.02), which also differed significantly from all other emotions, as well as a significant difference between anger (*M* = 1.20, *SD* = 0.56) and disgust (*M* = 1.02, *SD* = 0.13). The main effect of Sex was due to higher emotional ratings in females (*M* = 1.62, *SD* = 1.02) than in males (*M* = 1.52, *SD* = 0.85) (see Table 2 for means per group and emotion). The main effect of Time was due to overall higher ratings, that is, more intense emotions, at T3 (*M* = 1.58, *SD* = 0.95) than at T4 (*M* = 1.52, *SD* = 0.93).

**TABLE 2:** Overview of means of all physiological responses per group.

	**Control men (*N* = 14)**	**Control women (*N* = 16)**	**Threat men (*N* = 15)**	**Threat women (*N* = 16)**
Cortisol T2	5.87 (3.04)	3.58 (1.62)	6.92 (3.23)	5.03 (1.44)
Cortisol T3	6.02 (3.38)	2.98 (1.17)	5.81 (2.16)	4.78 (1.65)
Cortisol T4	5.23 (3.5)	2.72 (0.99)	4.63 (1.7)	4.45 (1.63)
HR T1	69.86 (10.04)	71.13 (9.37)	76.15 (13.56)	72.53 (7.78)
HR T2	70.57 (6.96)	70.75 (7.96)	76.4 (12.43)	74.94 (7.41)
HR T3	69 (6.75)	69.88 (10.45)	74.73 (14.65)	72 (7.37)
HR T4	69.21 (6.39)	69.25 (9.95)	69.73 (11.22)	72.88 (7.86)
HR T5	65.15 (8)	68.63 (8.16)	70 (12.07)	68.5 (7.53)
HR T6	65.5 (7.19)	65.88 (6.61)	69.93 (12.42)	67.56 (7.47)
SBP T1	130.07 (11.06)	105.38 (5.77)	125.62 (12.98)	110.47 (6.74)
SBP T2	124.93 (13.3)	104.94 (8.03)	119.73 (14.54)	110 (7.19)
SBP T3	119.07 (32.02)	105.44 (9.68)	119.67 (14.72)	108.81 (11.15)
SBP T4	124.43 (10.47)	104.88 (6.57)	117.13 (15.25)	107.19 (6.96)
SBP T5	121.08 (10.63)	102.94 (8.54)	119.53 (10.41)	105.63 (6.68)
SBP T6	122.36 (10.59)	103.56 (7.59)	115.07 (10.33)	105.44 (10.89)
DBP T1	72.36 (5.73)	70.31 (7.12)	69 (10.26)	68.8 (6.17)
DBP T2	68.71 (6.12)	68 (6.35)	67.47 (9.01)	68.25 (6.5)
DBP T3	71.79 (7.98)	68.56 (5.14)	66.6 (10.01)	69.13 (7.63)
DBP T4	69.43 (6.32)	68.81 (8.64)	64.67 (9.57)	68.94 (7.09)
DBP T5	67.92 (5.57)	66.94 (4.46)	64.4 (9.63)	66.06 (6.14)
DBP T6	69.5 (6.8)	67.75 (5.13)	66.73 (8.66)	67.38 (5.83)

*HR, heart rate; SBP, systolic blood pressure; DBP, diastolic blood pressure.*

Decomposing the two significant two-way interactions revealed higher ratings for anger (*p* ≤ 0.001) and surprise (*p* = 0.012) in the threat group than in the control group, along with overall higher ratings in the threat group than in the control group at T3 (*p* = 0.015), that is, after the first block of the VISTTA. These effects need to be viewed within the context of the Group ^*^ Emotion ^*^ Time interaction: For anger and surprise, ratings differed between threat and control only at T3 and T4 (*p* ≤ 0.002), not at T2 (*p* ≥ 0.068) (before the VISTTA). Moreover, ratings for happiness differed at T4, with lower ratings in the threat group than in the control group (*p* = 0.0026). Crucially, temporal changes of emotional ratings were limited to the threat group: Here, ratings for anger increased from T2 to T3 (*p* ≤ 0.001), and from T2 to T4 (*p* = 0.005). Similarly, ratings for surprise increased from T2 to T3 (*p* = 0.022). Analogously, happiness decreased from T2 to T4 (*p* ≤ 0.001), and T3 to T4 (*p* = 0.030), while no such changes over time were evident in the control group (*p* ≥ 0.0164) (Figures 3D–F).

The Group ^*^ Sex ^*^ Emotion interaction was due to sex-specific responses: In males, higher ratings in the threat than in the control group were evident for anger and surprise. In females, differences in emotional ratings between threat and control emerged for anger and happiness, with higher and lower values in the threat group, respectively. Comparing males and females directly indicated differences in the rating of happiness only for the control group, that is, females rated themselves as happier than males.

### Physiological Responses

#### Cortisol

The repeated measures ANOVA with Time as within-subject factor and Sex and Group as between-subjects factors showed a main effect of Time [*F*(1.322,75.930) = 24.031, *p* ≤ 0.001, $\eta_{\text{p}}^{2}$ = 0.297] with T2 ≥ T3 (*p* = 0.013), T3 ≥ T4 (*p* ≤ 0.001), and T2 ≥ T4 (*p* ≤ 0.001). We also found a main effect of Group [*F*(1,57) = 6.193, *p* = 0.016, $\eta_{\text{p}}^{2}$ = 0.098] with higher cortisol levels in the threat group (*M* = 5.25 nmol/L, *SD* = 1.88) than the control group (*M* = 4.31 nmol/L, *SD* = 2.58) at all three time points (*p* ≤ 0.036), and a main effect of Sex [*F*(1,57) = 9.051, *p* = 0.004, $\eta_{\text{p}}^{2}$ = 0.137], whereby men (*M* = 5.75, *SD* = 2.57) had higher cortisol levels than women (*M* = 3.92, *SD* = 1.58) at all three time points (*p* ≤ 0.034).

A significant three-way interaction between Time ^*^ Sex ^*^ Group emerged [*F*(1.332,75.930) = 3.653, *p* = 0.048, $\eta_{\text{p}}^{2}$ = 0.060]. *Post hoc* comparisons for men and women separately showed a main effect of Group among women [*F*(1,30) = 14.233, *p* = 0.001] with higher cortisol levels at all three time points in the threat group (*p* ≤ 0.027) (Figure 3C). Cortisol levels in men did not differ between groups (*p* ≥ 0.212). Comparing men and women in both groups showed a main effect of Sex in the control group [*F*(1,28) = 7.989, *p* = 0.009] with higher cortisol levels in men than women. Cortisol levels in the threat group did not differ between men and women (*p* ≥ 0.137) (see Table 2 for an overview of means and standard deviations). Time point comparisons to investigate the course of cortisol levels showed that the cortisol level did not change over time for women in the threat group (*p* ≥ 0.281). Women in the control group showed a significant decrease between T2−T3 and T2−T4 (*p* ≤ 0.040). The control group in men led to a decrease between T3 and T4 (*p* ≤ 0.001). In the threat group, cortisol levels decreased significantly between T2−T4 and T3−T4 (*p* ≤ 0.001). All other time point comparisons did not reach significance (*p* ≥ 0.116) (Figure 3C).

#### Heart Rate and Blood Pressure

The repeated measures ANOVA with Time as within-subject factor and Sex and Group as between-subjects factors showed a main effect of Time [*F*(3.919,207.718) = 18.127, *p* ≤ 0.001, $\eta_{\text{p}}^{2}$ = 0.255]. No main effects of or interactions with Group and Sex were found (*p* ≥ 0.224). The Time ^*^ Sex ^*^ Group interaction showed a trend toward significance (*p* = 0.053). The overall pattern showed that heart rate decreases in both groups, with more fluctuation in the threat group.

We found a main effect of Time for both systolic and diastolic blood pressure (SBP and BPD, respectively) (*p* ≤ 0.17), with a general decay over time. For SBP, there was a main effect of Sex, showing that men had higher SBP than women (*p* ≤ 0.022). No difference between sexes was found for DBP (*p* ≥ 0.795). Blood pressure did not differ between groups (*p* ≥ 0.239).

## Discussion

The aim of the current study was to develop and validate a new, fMRI compatible, social threat paradigm that implements a realistic representation of nowadays’ digital communication environments. As a second objective, we were interested if and how social-evaluative threat affects men and women differently. The opening sentences were created in a way that they stated something about the participant’s personality or interests, so that the reactions that followed from the interlocutors would directly target the participant. Reactions in the control group were of an agreeing and accepting nature so that those participants did not experience any social evaluative threat. Our results indicate that the VISTTA elicits both subjective, emotional, and physiological responses as apparent from lower willingness to cooperate after negative reactions, reactions rated as more negative in the threat group, increased feelings of anger and surprise, decreased feeling of happiness, negative mood decreased in the control group, but stayed stable in the threat group and the stable cortisol levels in women in the threat group throughout the experiment. However, increased physiological measures at the start of the testing session could reflect pre-experimental arousal, as the experiment started approximately 30 min after arrival.

### Relevance of VISTTA as New Social-Evaluative Threat Paradigm

Although the VISTTA contains obvious similarities with the Chat-room Task (Donate et al., 2017), these paradigms target different concepts. The Chat-room ostracizes participants by not asking them the same amount of questions as the confederates. This “lack of interest” in the participant is the driving force behind the ostracism induction. The content of the questions and answers does not play a role. The VISTTA is differently structured, whereby participants are continuously involved in the conversations. Our goal was to use personally directed rejection to drive the experience of social-evaluative threat. Participants might have felt ostracized during the VISTTA when the two confederates repeatedly agreed, and together disagreed/insulted the participants’ perspective.

### Subjective Responses

Lower willingness to cooperate after negative reactions than after positive ones shows rejection negatively affected the motivation for social, cooperative interactions. We did not find sex differences for this measure despite different characteristic coping strategies between men and women. Nickels and Kubicki (2017) reported that performance stress, induced via the TSST, led to less prosocial behavior in men and more cooperative behavior in women. The VISTTA is not performance based, which could be a possible explanation why this sex difference is not reflected in our findings. As an additional validation of social rejection, participants rated how positive or negative they experienced all reactions they received. As this was also a 1–5-Scale, just like “willingness to cooperate” the findings showed an almost identical pattern, with more negative ratings for negative comments and more positive ratings for positive comments. Men and women showed a similar rating pattern. Although these measures were very similar, we tried to target different concepts. “Willingness to cooperate” was hypothesized to reflect a motivation for facing the two individuals who rejected the participant, whereas “comment ratings” to reflect the level of positivity or negativity of each individual reaction. We wanted to indirectly measure social-evaluative threat using these measures.

### Affective Responses

Also, no sex difference emerged for the subjective mood ratings. We found that positive mood decreased over time for both men and women. This decrease, however, was seen in both the threat and control group, suggesting the negative comments during the VISTTA did not affect participant’s positive emotional state. Negative mood did differ between groups. Participants in the control group reported a decreased negative mood in the first half of the VISTTA. The threat group showed an increase, although that did not reach significance. These findings suggest that the inclusive interactions in the control condition positively affected the negative mood. This underlines that positive and negative mood are not bipolar, but rather change independently from one another. We also demonstrated effects on multiple emotions such as anger, surprise, and happiness. Over the course of the threat version of the VISTTA, participants reported increased feelings of anger and surprise, and decreased feelings of happiness. Similar results have been reported using other exclusion paradigms. Unfair exclusion, compared to fair exclusion in a modified Cyberball Task, was linked to increased anger (Chow et al., 2008). Exclusion from participation in the Chat-room Task also led to higher anger ratings (Donate et al., 2017). Our finding regarding surprise indicates that the negative interaction was unexpected, since the control group did not report any changes for this emotion. Decreased happiness in the threat group, contrary to stable happiness levels in the control group, indicates that the VISTTA negatively affected the positive state. Two factors might contribute to these findings. The most evident is the content of the personally directed negative comments that affects the emotional state of the participants. Second, the anticipation of having to face the interlocutors after the chat Task and to cooperate with them on a separate Task for additional monetary reward could contribute to a less positive mood.

### Physiological Responses

Contrary to our *a priori* hypotheses, the VISTTA did not induce cortisol and heart rate increases. A possible explanation is that there is no direct social evaluation, but indirect via a computerized communication. Other fMRI compatible paradigms, such as Cyberball (Williams et al., 2000) have also been found to not elicit a cortisol increase in both men and women (Zöller et al., 2010; Zwolinski, 2012; Seidel et al., 2013; Gaffey and Wirth, 2014; Radke et al., 2018). Cortisol increases are generally found after a stressor that includes direct personal interaction. Using a modified version of the TSST, Woody et al. (2018) investigated the effect of social-evaluative threat, and added cognitive load as additional factor of interest. They reported increased cortisol and blood pressure in response to social-evaluative threat, but a flat line for the non-social-evaluative threat group. Following the circadian rhythm, in healthy individuals, cortisol levels peak early in the morning and, without a stressor, decline throughout the day (Krieger et al., 1971; Weitzman et al., 1971; Debono et al., 2009; Chan and Debono, 2010). The fact that we do not see this decline in women in the threat group during the experiment could be an indication that social-evaluative threat by the VISTTA elicits an endocrinological response in women. For men, the decline in cortisol, as well as in heart rate, might indicate that they habituate to the social evaluation. As the social evaluation occurs through a computer without face-to-face interaction, the physiological responses we found could be dampened due to a more indirect threat. During the YIPS, female, but not male, participants, who are excluded during the course of a conversation with two confederates, show a cortisol increase (Stroud et al., 2000, 2002; Zwolinski, 2008). However, this cortisol response is not replicated in all studies (Linnen et al., 2012). Women appear to respond more strongly to social rejection, whereas men show increased cortisol responses to achievement challenges (Stroud et al., 2002; Kogler et al., 2017). In the study of Blackhart et al. (2007) participants were told that no one wanted to be paired with them to complete a Task after a group interaction session. Although the rejection did not come directly from the confederates, cortisol levels were significantly higher following social rejection compared to acceptance. It seems that direct personal interaction whereby investigators/jury/peers judge or reject participants is an important factor to elicit a cortisol increase, and that it is particularly effective in women. Meeting the two confederates could help reinforce the cover story and elicit a stronger response to rejection. Looking back at the factors influencing the stress response that we discussed in the section “Introduction,” an important note here is that the above-mentioned studies either included women not using OC or did not report on contraceptive use. Also, to this day, the majority of research papers focuses on sex difference whereas gender identity has been shown to differently affect the stress response. It would be a valuable addition to future research to assess not only sex but also gender, and include it as a factor of interest or at least as confounding factor.

At the start of the experiment, heart rate was significantly higher for both men and women compared to when the VISTTA was finished; however, this was seen in both experimental groups (threat vs. control). This decline opposes our previous hypothesis that HR increases as an effect of the social threat. Throughout the entire experiment, SBP was significantly higher for men compared to women, with no effect of experimental group. Men have, in general, a higher SBP than women (Reckelhoff, 2001). Overall, the VISTTA did not elicit a significant response in HR or blood pressure.

### Limitations

We were unfortunately not able to measure a continuous heart rate signal or skin conductance. The blood pressure monitor we used had to be attached separately for each time point and hence only enabled us to acquire heart rate and blood pressure for T1–T6. It was therefore not possible to directly compare physiological responses to negative and positive feedback, only between-subjects. During an acute stressor, the release of catecholamines increases heart rate and blood pressure (McEwen, 2007). Heart rate is therefore a suitable measure to investigate the immediate response to a stressful situation. To directly compare the effects of negative and positive feedback, a continuous heart rate signal would shed more light on the physiological responses to social-evaluative threat and be a suitable indicator for stress response. Although physiological measures could not be acquired continuously, we did have a behavioral measure after every conversation, allowing us to directly compare subjective effects of positive vs. negative feedback.

For this study, we chose to test a group with a specific age and social background to restrict possible confounding factors. This might result in lower generalizability of the results. Also, given this is the initial study investigating the effects of VISTTA, future studies including larger and different samples should shed more light on wider applicability of this new paradigm. Since all conversations were in German, the VISTTA should be adapted for other studies using non-German speakers. All opening sentences and responses are also available in English. Also, two topics should be changed, as they were specific for the region the study took place.

We made the choice to only include women on OCs to control for hormonal fluctuations and interpersonal differences in the menstrual cycle. It should be noted that OCs heighten estrogen levels and consequently dampen the stress response in women (Pruessner, 2018).

Although, we included a variety of affective measurements, additional measurements such as feelings of embarrassment and changes in self-esteem could have served as extra validation for experiencing social-evaluative threat. Validation of the VISTTA by means of psychological responses was aimed at differences in positive and negative mood, as well as changes in a range of emotions including anger, fear, happiness, and surprise among others. We did not find changes in fear and sadness, which may suggest participants experienced surprise rather than social-evaluative threat from the interactions. Increases in anger, however, are comparable to other studies investigating social exclusion using the Cyberball and Chat-room Task (Chow et al., 2008; Donate et al., 2017).

## Conclusion and Future Directions

Implementing personally directed, verbal negative feedback, we applied the VISTTA to induce social-evaluative threat. Men and women in the threat group responded similar on the subjective level, that is, with increased anger and surprise and a lower willingness to cooperate in comparison to the control group. However, physiological measures differed between both groups and sexes. We demonstrated an overall higher endocrinological response in the threat group. Regardless of group, a cortisol decay over time was reported for men, whereas women showed a stable cortisol level over time in the threat group and a decay in the control group. These findings might indicate stronger habituation in men than in women and underline the importance of multi-level assessment of responses to social-evaluative threat, even in computer-mediated communication. Further replication and validation during fMRI will be crucial to determine its effects in different experimental settings. It will be of interest to which extent meeting the interlocutors affects the perception of social threat. Also rating how much the participants identify with the opening statement of choice could give more insight how threatening the negative reactions might be perceived. Social media environments, as used in the VISTTA, can lower the threshold for negative interaction, which, in turn, can elicit feelings of stress and rejection. Given the increasing influence of online communication platforms, the VISTTA is a useful addition for research on social-evaluative threat and psychosocial stress. In reaction to performance and evaluative stressors, sex and gender have been shown to affect the stress response differently (Pruessner, 2018). As most research has focused on the role of sex to differentiate between males and females, gender has not been given the same level of investigation despite having a demonstrated effect. The discussion on sex and gender has taken a flight over the last few years, both in society, and in the scientific community. The VISTTA enables multi-level assessment of social-evaluative threat, hence, using samples with varying compositions of sex and gender identity, this paradigm could help bridge the gap between sex and gender in this particular field.

## Ethics Statement

The experimental protocol was carried out in accordance with the provisions of the World Medical Association Declaration of Helsinki. All subjects gave written informed consent. The protocol was approved by the local ethics committee at the Medical Faculty of RWTH Aachen University.

## Author Contributions

ST created the first draft, finalized the manuscript, and performed the data collection and analyses. UH and TA took part in interpreting the data and revision of the manuscript. BD and SR conceived and designed the project, and took part in interpretation of the data and revision of the manuscript.

## Conflict of Interest Statement

The authors declare that the research was conducted in the absence of any commercial or financial relationships that could be construed as a potential conflict of interest.
