# Supplementary material for: The Verbal Interaction Social Threat Task: A New Paradigm Investigating the Effects of Social Rejection in Men and Women
Source: Front Neurosci. 2019 Aug 7;13:830. doi: 10.3389/fnins.2019.00830 (PMC6692967; doi:10.3389/fnins.2019.00830)
Supplement: Supplementary file 1 [file Data_Sheet_1.docx]

***Supplementary Material***

**The Verbal Interaction Social Threat Task: a New Paradigm Investigating the Effects of Social Rejection in Men and Women**

**Sanne Tops^1^, Ute Habel^1, 2^, Ted Abel^3^, Birgit Derntl^4^, Sina Radke^1, 2*^**

^1^ Department of Psychiatry, Psychotherapy and Psychosomatics, Faculty of Medicine, RWTH Aachen, Germany

^2^ Jülich Aachen Research Alliance (JARA) – BRAIN Institute I: Brain Structure-Function Relationships: Decoding the Human Brain at systemic levels, Research Center Jülich GmbH and RWTH Aachen University, Jülich/Aachen, Germany

^3^ Iowa Neuroscience Institute, Department of Molecular Physiology and Biophysics, Carver College of Medicine, University of Iowa, Iowa City, USA

^4^ Department of Psychiatry and Psychotherapy, Medical School, University of Tübingen, Tübingen, Germany

*** Correspondence:**Sina Radke
sradke@ukaachen.de

**Supplementary Table 1. Overview of all opening sentences and matching reactions from the interlocutors.** Topics that are marked green contain positive reactions, whereas red marked topics contain negative reactions. ++ indicates that a topic contains very negative reactions.

| Opening statement participant | Reply from confederate participants (a: Julia, b: Daniel) |
| --- | --- |
| **Aachen** |  |
| 1) I like the old city center, especially the Aachener Dome | 1a) Indeed, the historical center is very nice.  1b) I like to walk through the city. |
| 2) I like to go to the Pontstraße with all the cafes and restaurants | 2a) The Pontstraße is nice, I like to go there with my friends.  2b) I like to go out for dinner there sometimes. |
| 3) I like to go shopping, there is a great diversity of shops in the city center | 3a) Aachen is indeed nice for shopping.  3b) I only like it when I’m looking for something specific. |
| 4) I don’t like Aachen very much, but moved here to pursue my study | 4a) I think Aachen is quite small, I prefer bigger cities like Köln, Hamburg and Berlin.  4b) I moved here for my study too, but I’m not really a city person, I rather live in a small town. |
| **Sport** |  |
| 1) I play a team sport like football, handball or basketball | 1a) I don’t like team sports, I prefer to go running.  1b) I think team sports are more like a game than a real sport. |
| 2) I don’t like to play sports | 2a) How can you not like sports, are you lazy?  2b) I often go to the gym, I actually get a lot of energy from working out. |
| 3) I play an individual sport like tennis or athletics | 3a) Team sports are so much more fun than sporting on your own.  3b) Individual sports are lame, I really like to play a team sport, especially football. |
| 4) I like to watch sports on TV | 4a) I don’t watch sports on TV, I prefer to play sports myself.  4b) You don’t get fit by just watching sports! |
| **Spare time** |  |
| 1) In my spare time I like to play/listen to music | 1b) I don’t play an instrument, I like to play sports when I am free.  1a) Making music is not really my thing, I prefer to hang out with my friends in my spare time. |
| 2) In my spare time I like to hang out with my friends | 2b) I see my friends every day at the university, when I am free I spend time with my girlfriend.  2a) I agree, but I like to spend some time by myself or I go into town to go shopping. |
| 3) In my spare time I visit my family | 3b) I don’t go to my family often, I prefer to stay in Aachen and meet my friends.  3a) My family lives too far away, therefore I can’t visit them a lot. |
| 4) In my spare time I go into town | 4b) I really prefer to go into nature instead of staying in the city.  4a) I like to play sports when I am free, or hang out with my friends. |
| **Moving out** |  |
| 1) I think it is exciting to live on my own, away from my parents | 1a) Was it really that bad to live with them?  1b) When I was still living at home, I didn’t have to do all the household tasks, now I have to cook, clean and do the laundry myself. I liked to live with my parents and siblings. |
| 2) Living with roommates is a lot of fun | 2a) I don’t think so, I would rather have my own place instead of sharing everything with roommates.  2b) I share a flat with one other person, I wouldn’t like living in a typical WG. |
| 3) I am a bit annoyed by my roommates, they are too loud, make a mess, don’t clean | 3a) I really enjoy living in a WG, and I don’t get upset by every little thing.  3b) I am a bit messy myself, so I don’t mind if it is not spotlessly clean. |
| 4) I live together with my boyfriend/girlfriend | 4a) I don’t have a boyfriend, but I would not want to move in together so early.  4b) I would not want to live together with my girlfriend yet, I just really enjoy my freedom. |
| **Study** |  |
| 1) I study a lot, because I want to have good grades | 1b) Grades are not the most important thing, don’t you have a social life?  1a) I like to have good grades, but I don’t sacrifice my social life just for grades. |
| 2) I like to study together with fellow students | 2b) I don’t like to study in a group, I can concentrate much better if I study by myself.  2a) That is not very effective, you get a lot more work done if you study by yourself. |
| 3) I often don’t go to the lectures, I go through the study material myself | 3b) You get a lot of extra info during the lectures, so it’s quite dumb not to go there.  3a) I think lectures are nice, and do you really have the discipline to go through everything yourself? |
| 4) I think the workload of my study is very high and it is difficult to keep up | 4b) My study hasn’t been difficult for me at all, the workload is actually not too bad.  4a) So far I haven’t had any problems either, everything is relatively easy for me. |
| **Mensa** |  |
| 1) I think the mensa is great, they offer a large variety of food and for a low price | 1b) The mensa is not great at all, it is always crowded and I also don’t like the food.  1a) I go there, but I prefer home cooked meals. |
| 2) I think the mensa is too crowded, you have to wait very long to get your food | 2b) You’re just whining, I like going to the mensa, the food is good and quite cheap.  2a)It is not that bad to wait a bit, just be patient!! |
| 3) I prefer to bring my own lunch, I don’t like the food in the mensa | 3b) Bringing your own lunch is such a hassle, and I actually like the food in the mensa.  3a) I think so too, I wouldn’t want to prepare my meals in the morning, I’d rather sleep longer. |
| 4) I prefer to go somewhere else for lunch, like cafés, lunchrooms or fast-food restaurants | 4b) Why would you do that? The mensa is just fine.  4a) I do like the mensa, and you don’t have the hassle to go someplace else. |
| **Job** |  |
| 1) I have a side job to pay for my study | 1a) Do you even have a social live, when you’re either studying or working?  1b) My parents pay for my study so I don’t have to work. |
| 2) I don’t have a side job because I don’t need the money | 2a) I have to pay for my study myself, I can’t afford not to have a side job.  2b) Your parents probably pay for everything, but I really need my job to make ends meet. |
| 3) I took a side job here because I want to meet a lot of new people | 3a) You need a job for that? Don’t you have a social life then?  3b) I already have plenty of friends, I really don’t need much more. |
| 4) My study consumes most of my time so I don’t have time for a side job | 4a) You should just plan everything better.  4b) I have enough time, I think I just manage my time better. |
| **Going out** |  |
| 1) I like to go out as often as possible | 1b) Nightclubs are not my thing, I don’t like to go out.  1a) Going out is overrated, I prefer to spend some real time with my friends. |
| 2) I don’t like going out very much, I prefer to stay at home and watch TV | 2b) I do like to go out, I think staying at home is quite boring.  2a) Staying home and watch TV, how dull is that! |
| 3) I sometimes go into town to have a few drinks with my friends | 3b) I like to go out, I think staying at home is quite boring.  3a) You should go to a club, that’s much cooler than sitting in a café. |
| 4) Instead of going out, my friends come over or I go to their place | 4b) I like to go out, I think staying at home is quite boring.  4a) You should go to a club, sitting at home is just dull! |
| **Family** |  |
| 1) I don’t like it that I’m so far away from my family, I would like to see them more often | 1a) I don’t mind being away from them, this way I can really live my own life.  1b) I spend my weekends in Aachen anyways too, even though my parents don’t live very far away. |
| 2) My family lives close to Aachen and I visit them as often as I can | 2a) I go home every once in a while, but I prefer to spend my weekends here.  2b) I have my own life here now, so I usually stay in Aachen. |
| 3) I don’t have a great bond with my family and I don’t go home very often | 3a) I do go home often, I like to visit my family.  3b) I go home as often as I can, I have a big family and we always have a great time together. |
| 4) I don’t go home often, I like to spend my weekends hanging out with my friends here in Aachen | 4a) I still have a lot of friends who live close to my parents, and I like to see my family as well.  4b) So do I, I still have a great social life back home. |
| **Travelling** |  |
| 1) When I am travelling I like to explore cities and get to know other cultures | 1b) I often go on a city trip, I really like seeing all these famous places.  1a) Yes me too, I have seen quite some cities already. |
| 2) I like to go on a beach vacation | 2b) When I am on holiday I usually just want to chill and sometimes swim in the sea.  2a) I really like going to the beach, just relax and enjoying the sun. |
| 3) When I go on vacation I often go camping and enjoy nature | 3b) I also like to go camping, especially because you have the freedom to stay wherever you want.  3a) We often go to Austria and walk in the Alps in summer holiday, I just enjoy being outside. |
| 4) I like to go on active vacations, like going hiking, sailing, snorkeling or climbing | 4b) I like active holidays so much more than just going to the beach or do cultural stuff.  4a) So do I, I really like to go sailing. Being out on the sea sailing from one place to another is just awesome. |
| **Gaming++** |  |
| 1) I like to play games like WoW, League of legends, GTA, Skyrim etc. | 1a) Really? I don’t like gaming at all, I think it is a waste of time.  1b) I don’t like to play online games, I know better ways to spend my time. |
| 2) I don’t like to play games, I can do something better with my free time | 2a) I think you’re wrong about that. Playing games is a lot of fun, especially if you play with your friends.  2b) I am really in the online gaming, how can you not like that, it’s awesome. |
| 3) I like to play board games like Chess, Monopoly, The settlers of Catan, Risk, Munchkin etc. | 3a) That is boring, who still plays these games anyway.  3b) I think online gaming is a lot more fun, board games are just dull. |
| 4) I sometimes play computer games, but not very often | 4a) I don’t like computer games at all, I never ever play them, only nerds play those kinds of games.  4b) I don’t like to play computer games, I know better ways to spend my time. |
| **Books** |  |
| 1) I really enjoy reading books, and I often have a book on my nightstand | 1b) That is just dull, don’t you have anything better to do?  1a) I don’t like reading either, I think reading a lot is quite nerdy. |
| 2) I read the textbooks for my study but besides that I don’t like reading very much | 2b) Reading is important for your development, it’s dumb not to invest in that.  2a) You actually read the textbooks? I don’t know anyone who still does that. |
| 3) I read during the holidays, but during the semester I’m mostly occupied with my study | 3b) That’s just bullshit, you can make time for it.  3a) I agree, reading is important and it doesn’t have to take too much time. You could just read before going to bed. |
| 4) I sometimes read, but only when I have a really good book | 4b) You don’t know beforehand if a book is good, so you can just as well read more books and put them away if they’re not good.  4a) That’s just a lame excuse not to read. |
| **Food/cooking** |  |
| 1) I’m not very good at cooking, but I like it and I try to make something fresh as often as I can | 1b) I can’t cook either, usually my roommate cooks.  1a) My cooking skills are trial and error too, but I would like to be better at it. |
| 2) One of my roommates usually cooks or I eat a microwave meal, I don’t like cooking very much | 2b) Either my roommate cooks or I have take-out or pizza or something.  2a) I don’t like cooking either, I usually prepare something easy. |
| 3) I like cooking, but I have a warm meal at the mensa, so when I get home I eat something easy like bread or soup etc. so I don’t have to cook very often | 3b) It indeed saves time and money and I like that you can sit down and have lunch with friends.  3a) Yeah so do I, it’s just convenient and you don’t have to do all the groceries. |
| 4) I really enjoy cooking and I often try new recipes | 4b) Good for you! I wish I could cook, but unfortunately I am not really a chef.  4a) I like to experiment a lot too, especially when it turns out great. |
| **Environment++** |  |
| 1) I am aware of the environment and I try to contribute by, for example, separating garbage and bike instead of taking the car. I think all little bits help | 1b) It’s quite naïve to think that by just separating your garbage you can make a difference.  1a) I don’t really care, on such a small scale you can’t contribute anyways. |
| 2) I am not very interested in the environment, I think there is a lot of fuss about global warming and exhausting raw materials, but I think there will be a solution in time | 2b) How can you not be interested in that? If everybody thinks like you this planet will be destroyed even sooner.  2a) We only have one planet, global warming and exhausting resources really are big problems. It’s naïve to think that everything will be sorted anyway. |
| 3) I think the environment is a political issue and should be handled globally. I don’t think that I can make much of a difference myself | 3b) Well if everybody thinks like you than we’ll destroy this planet even sooner.  3a) If you don’t take any responsibility, then indeed nothing will change! |
| 4) I don’t think that I can make much of a difference on such a small scale | 4b) Well if everybody would be so careless about the environment then there will never be an improvement.  4a) Yeah I agree, it would help if you would take some responsibility and don’t add to deterioration of the world. |
| **Shopping** |  |
| 1) I like to go shopping in the city center because there is a large variety of shops | 1a) Shopping online is so much easier, and there is even a larger offer of brands.  1b) I hate shopping, I don’t see why people actually like it. |
| 2) I prefer to order online, it’s a lot easier and you don’t have to leave the house | 2a) Ordering online is lame, and lazy. How much effort is it to just go into town? And then the retail owners can keep up their business.  2b) I don’t like shopping in any way, not online and also not in actual shops. |
| 3) I like to go to a big mall where all the shops are close together | 3a) Big malls are so overrated, shopping is no fun at all there.  3b) I hate shopping, I don’t see why people actually like it. |
| 4) I don’t like to go shopping at all. It’s just so boring | 4a) It is not boring at all! I really like to go shopping when I am free.  4b) Even as a guy, I sometimes like to go into town and buy some new stuff, it’s really not that boring. |
| **Transport** |  |
| 1) I bike as often as I can instead of taking the car, I like being outside and with biking I’m active too | 1a) Here in Aachen it’s raining all the time and biking is not very efficient with all the hills.  1b) The bus is a lot more convenient than biking, and you stay dry. |
| 2) I often take the car, I don’t really like biking, especially in Aachen with all the hills | 2a) That’s just being lazy. Biking is healthier and better for the environment.  2b) I agree with that, the hills in Aachen are not even high. |
| 3) I usually take the bus because it’s free for students, I don’t like to bike or walk all the time | 3a) That’s just lazy, biking is free too.  3b) and it is even easier, and you don’t have to change busses all the time. |
| 4) If the distance is not too long, I like to walk, especially if the weather is nice | 4a) Walking takes way too long, I would either bike or take the bus.  4b) Don’t you have anything better to do? I always travel as fast as possible. |
| **Animals/pets** |  |
| 1) We always had pets at home, I really like having animals around and I think I’ll have pets after I graduate | 1a) My brother is allergic, so therefore we only have a goldfish. But I would have liked having more pets.  1b) We have a dog named Balu, I cannot imagine not having pets around. |
| 2) I like animals, but I wouldn’t want to have pets, it is just too much work | 2a) I would consider taking a small pet, like a rabbit or something, that doesn’t take too much work.  2b) I like animals too, but I don’t want to have them in my house. |
| 3) I am not a big fan of animals, and I don’t want to have pets in my house | 3a) Me neither, and I don’t want to limit my freedom because I have to look after an animal.  3b) I would consider having a small pet, but only if it doesn’t consume too much time. I’m not a zookeeper :P |
| 4) I would like to have some farm animals in the future, like chickens, goats, sheep or ponies etc. | 4a) That would be very cool, but probably not very realistic ☺  4b) I definitely want to have animals in the future, but I think these are a bit too big for me. |
| **Religion** |  |
| 1) Religion plays an important role in my life | 1a) Then you must be the only one, I don’t know anyone my age who is religious.  1b) I am not religious at all, I believe more in science |
| 2) I think everyone is entitled to believe what they want, as long as they keep it to themselves. | 2a) That is very disrespectful. Every religion has its own rituals and customs and you should respect that.  2b) I agree, everyone should be able to express their religion the way they want. |
| 3) I am not religious, I don’t believe in a God at all | 3a) You really think that this is it and there’s nothing more between heaven and earth?  3b) I just find that hard to believe. |
| 4) I think most religions share similar core values and beliefs | 4a) That’s bullshit! Especially Christianity and the Islam are not even closely related!  4b) I agree, if all religions were similar then there wouldn’t be so many wars. |
| **Movies** |  |
| 1) I really like to watch action movies and I recently saw the latest James Bond | 1a) Action movies can be fun, but only if there are not too many special effects. Then it easily becomes unbelievable.  1b) I like a good action movie ones in a while too. |
| 2) I’m into fantasy/science fiction, I’m excited about the new Star Wars movie | 2a) My personal favorite is the Hunger Games! I can’t get enough of it.  2b) Then we have that in common. Sci-Fi is my guilty pleasure ☺ |
| 3) I don’t watch a lot of movies, I prefer series or documentaries | 3a) I think movies are overrated. They are often predictable and similar to each other.  3b) Yes, I think so too. In a series you can have more complex characters and storylines. |
| 4) I like comedies, it’s always good to have a good laugh | 4a) Comedies are my favorite!  4b) I don’t watch a lot of movies, but I like a comedy every ones in a while. |
| **Music++** |  |
| 1) I really like listening to music, I use spotify/itunes/etc.to stream my favorite music | 1b) You actually pay to stream music when you can just download it? That’s just dumb!  1a) I am not very much into music, but I would definitely not pay for music, just turn the radio on! |
| 2) I don’t listen to a lot of music, but I sometimes turn on the radio and then I also hear news updates, which is nice | 2b) That is just lame, you can’t even decide what music you want to listen to.  2a) Music is great, how can you not be into it? |
| 3) I like to go to music concerts, especially from my favorite band/artists | 3b) Concerts are so overrated, you can’t see the artists well and the sound is also not that great.  3a) Yeah I agree with that, and definitely not worth the amount of money you have to pay for the tickets. |
| 4) I don’t like the popular music, I’m more fond of classical music | 4b) Classical music is boring as hell. Only old people still listen to that.  4a) I agree that is dull, there is so much cool music and you keep listening to that classical stuff. |
| **Social media++** |  |
| 1) I have several social media accounts, I’m on Facebook, Twitter and Instagram | 1a) I think it’s a waste of time, everyone just posts bullshit.  1b) I only have a Facebook account to communicate with fellow students. Twitter and Instagram are especially useless. |
| 2) I don’t have a social media account, if I need to talk to someone, I just text or call them or go by in person | 2a) Social media is now the most important way to communicate. You dwell in the past if you don’t adjust.  2b) That is what my grandparents do, that’s something from the old days. |
| 3) I have a Facebook account, but I don’t post anything very often | 3a) So you just have an account to snoop around at other peoples’ accounts?  3b) Why do you even have it if you don’t use it? |
| 4) I think social media is a great way to reach a lot of people and it comes in handy when discussing study related things with my fellow students | 4a) Are you the kind of person who puts everything on Facebook? I always find those people very annoying.  4b) I don’t see the point of using social media for your study. You see your fellow students every day. |
| **Future life++** |  |
| 1) I would like to have a job where I can earn a lot of money, and maybe have a family | 1b) You actually think money is the most important thing in life? That is just lame!  1a) You really need to get your priorities sorted! |
| 2) I want to settle down and have a family, money is not very important | 2b) What are you even studying for if you don’t have the ambition to build a career for yourself?  2a) How are you planning to support your family, do you want to let your kids grow up in poverty? |
| 3) I want to travel a lot, and maybe also work abroad | 3b) That doesn’t really sound like you have a plan.  3a) You have to grow up some time, you can’t keep playing around. |
| 4) I want to pursue a great career, I don’t think I want to have kids | 4b) There is more to life than just work!  4a) I just can’t understand why anyone would prefer a career over kids. |
| **Used media for news** |  |
| 1) I watch the news often on television | 1a) I do that too, I think it’s important to know what is going on in the world.  1b) I’ve been so busy that I haven’t seen much of the news lately, but usually I stay up to date. |
| 2) I don’t watch the news, I prefer to watch series or movies. I’ll hear about the most important news items anyway | 2a) I sometimes watch the news, but like you said the most important things can’t be ignored.  2b) I’m into movies too. When I have time to watch TV I usually watch a movie instead of the news. |
| 3) I have an app on my smartphone that I use to check the news | 3a) Yeah me too, I like it that you can check it whenever you want.  3b) I don’t have a smartphone, but otherwise I would definitely use the app too. |
| 4) In our WG we have a newspaper that I often read, or I listen to the radio | 4a) You have a newspaper in your WG? Nice!  4b) Having a newspaper is indeed nice, I usually use my phone to check the news. |
| **Future career** |  |
| 1) I don’t want to work for a boss, I would like to start my own business | 1b) I’m not in a commercial area, but I think it would be great being your own boss.  1a) Would be cool to be independent, but maybe also a bit risky. |
| 2) I don’t know exactly what I want to do after my study | 2b) My study is quite broad, so I also don’t know where I’m going to end up.  2a) Neither do I, I just picked a study that I found interesting. |
| 3) I want to be an engineer and design/ produce new products | 3b) It’s good that you already have a vision, I’m not sure yet what I want to do after college.  3a) Sounds cool, but isn’t being an engineer quite difficult. |
| 4) I would like to have a career in research, but I don’t know in which field exactly | 4b) Me too, I am studying biology and would like to do a PhD.  4a) I’m not in research, but it would be cool if you make new discoveries. |
| **Growing up** |  |
| 1) School always has been the most important thing when I was growing up. | 1a) That sounds so boring! Did you actually get to enjoy your childhood?  1b) I’m glad that I had a more care free childhood, There is more in life than just school. |
| 2) I have had quite a strict upbringing with a lot of discipline | 2a) That doesn’t sound like a lot of fun.  2b) I am glad I didn’t have a childhood like that. We were led free to do whatever we wanted and I turned out to be just fine. |
| 3) Because high school was shortened with a year, I found the last few years in high school to be quite stressful | 3a) I had the same, but it wasn’t stressful at all!  3b) Really? Stressful? For me it wasn’t stressful either, in other countries it is normal to go to school until 18yrs. |
| 4) I have had quite a free upbringing, there were not a lot of boundaries and I was let free to develop myself | 4a) That’s not really a proper upbringing. Kids need rules and regularity!  4b) Yeah I agree, not giving them boundaries won’t do them any good. |
| **In the library** |  |
| 1) I often go to the library to study, there I can concentrate better than at home | 1b) The library is so boring, it’s like a nerd club.  1a) Are you one of these people who are always studying and having no social life? |
| 2) I never go to the library, I rather study at home | 2b) The library really is the best to study, at home you just get distracted.  2a) Studying at home is so boring, and you can’t discuss problems with fellow students. |
| 3) I sometimes go there, but I think it is often too crowded and then I get easily distracted by others | 3b) You’re just wining, there is enough room if you go a bit early and you can wear headphones so you don’t hear other people.  3a) If you can’t even concentrate in the library, where can you? |
| 4) I don’t go to the library, only nerds go there | 4b) I don’t think you’ve ever been in the library, because that is absolutely not true.  4a) Yeah exactly, a lot of students go to the library, not only nerds. |
| **In the gym** |  |
| 1) I go to the gym quite often, you can really focus on specific muscles | 1b) I’ve been going to the gym for years, I really like it. 1a) I usually don’t go to the gym, but it’s good that you can go whenever you want, even when it’s raining. |
| 2) I never go to the gym, I prefer to do real sports | 2b) I do like the gym, but everyone has his own preferences.  2a) I don’t go to the gym either, I want to have a goal and not just pull up some weights. |
| 3) I like to go to the gym, but not during peak hours, it’s just too crowded then | 3b) Me too, I like to focus on my workout but I can’t when there are too many people.  3a) I usually don’t go to the gym, but when it’s so crowded I wouldn’t go either if I were you. |
| 4) I don’t like to go to the gym, I don’t feel very confident | 4b) Don’t care what others would think, if you want to go to the gym, just go.  4a) There are not only bodybuilders in the gym. Don’t let your insecurities hold you back. |
| **Politics++** |  |
| 1) I am very interested in politics, I think it’s important to know who makes the policies and to have some insight in the policies themselves | 1b) Politics are so boring. I really don’t want to waste time thinking about that.  1a) What’s the point of it, you can’t influence it anyway. |
| 2) During elections I inform myself about the different parties and their election points, but besides that I don’t have a particular interest in politics | 2b) That’s just useless, they often let go of these points anyways.  2a) I don’t think you have any influence on it so why would you even bother. |
| 3) I don’t care much about politics, it’s complicated and often not very transparent | 3b) If you’d show a little interest you’d see it is not complicated at all!  3a) That is absolutely not true, but you don’t know that since you don’t care! |
| 4) I really don’t like politics, they talk a lot but there are often no concrete measures | 4b) How can you say that?! Not all political decisions are national news. I thought you’d know that, but apparently not…  4a) That is absolutely not true, but you don’t know that since you’re not interested! |
| **Weather** |  |
| 1) I really enjoy summer, I like it when it’s warm | 1a) Yes me too, I just adore the sun!  1b) I don’t really mind, as long as it is not too hot it is fine with me. |
| 2) I prefer winter, especially when there is snow. | 2a) Snow is great! Then I just feel like a child again.  2b) I am more of a winter person too, I can’t handle high temperatures well. |
| 3) I can really enjoy rough, stormy weather | 3a) Really? Me too! But only if I am inside ☺  3b) I do like strong wind, but the rain not so much |
| 4) I don’t really care about the weather, I only don’t like it when it’s raining. | 4a) Everyone always complains about the weather, but I don’t think it’s that bad.  4b) I don’t mind either, not even when it’s raining. |
| **Carnaval** |  |
| 1) I really like Carnaval, I often go to the main cities where it’s celebrated like Cologne, Aachen, Düsseldorf and Bonn | 1b) Carnaval is one of my favorite holidays, but I rather celebrate it in my home town where I know a lot of people.  1a) You should try out Carnaval in Cologne, it’s the best! |
| 2) I never celebrated before, but this year is going to be the first time, I’m very excited | 2b) Carnaval is great, I think you’ll like it.  2a) I really grew up with Carnaval, it’s the best! |
| 3) I don’t like Carnaval, it’s just nothing for me | 3b) It’s all a bit too weird for me too.  3a) I rather go to a real club, I really don’t like the Carnaval music. |
| 4) I like Carnaval, but I don’t celebrate all the days | 4b) Carnaval is my favorite holiday, I always put a lot of effort in my outfit.  4a) You should try out Carnaval in Cologne, it’s the best! |

**Supplementary Table 2**. Trait measures (means and SD) of the men and women in the threat and control group. STAI(T) = State-Trait Anxiety Inventory (Trait scale); CISS = Coping Inventory for Stressful Situations with the subscales task (task-oriented coping), emotion (emotion-oriented coping) and avoidance (avoidance-oriented coping); PASA = Primary appraisal Secondary appraisal; RSQ = Rejection Sensitivity Questionnaire; SVF = Stressverarbeitungsfragebogen, i.e., stress coping with the subscales pos (positive coping) and neg (negative coping); WST = Wortschatztest, i.e., verbal intelligence

|  | Control  Men  N=14 | Control  Women  N=16 | Threat  Men  N=15 | Threat  Women  N=16 |
| --- | --- | --- | --- | --- |
| STAIT(T) | 49.43 (3.41) | 46.38 (2.22) | 47.87 (3.11) | 46.63 (2.42) |
| CISS_task | 30.29 (4.21) | 27.88 (5.04) | 30.80 (4.69) | 28.56 (5.51) |
| CISS_emotion | 22.79 (4.53) | 23.75 (4.48) | 19.67 (5.46) | 21.13 (6.25) |
| CISS_avoidance | 20.07 (6.02) | 25.88 (5.30) | 20.93 (4.13) | 23.81 (5.13) |
| Liebowitz_anxiety | 36.75 (5.93) | 42.69 (10.74) | 36.13 (6.08) | 38.38 (9.65) |
| Liebowitz_avoidance | 41.08 (10.20) | 42.56 (10.43) | 39.53 (8.09) | 34.88 (7.59) |
| PASA_stressindex | 0.54 (0.21) | 0.55 (0.19) | 0.51 (0.16) | 0.59 (0.19) |
| RSQ | 11.40 (3.38) | 10.08 (2.61) | 9.67 (3.41) | 8.47 (3.62) |
| SVFpos | 13.78 (2.32) | 12.98 (2.57) | 11.88 (4.88) | 12.72 (1.87) |
| SVFneg | 10.00 (2.40) | 9.16 (1.74) | 10.76 (3.10) | 8.94 (4.63) |
| Social Network Size | 22.50 (15.15) | 22.19 (10.06) | 24.27 (12.55) | 26.75 (9.89) |
| WST | 31.21 (3.77) | 26.94 (6.34) | 28.40 (5.36) | 30.06 (3.38) |
